# Hyperexcitable and immature-like neuronal activity in the auditory cortex of adult rats lacking the language-linked *CNTNAP2* gene

**DOI:** 10.1093/cercor/bhab517

**Published:** 2022-02-01

**Authors:** Kaela E Scott, Rajkamalpreet S Mann, Ashley L Schormans, Susanne Schmid, Brian L Allman

**Affiliations:** Department of Anatomy and Cell Biology, Schulich School of Medicine and Dentistry, University of Western Ontario, London, ON, Canada; Department of Anatomy and Cell Biology, Schulich School of Medicine and Dentistry, University of Western Ontario, London, ON, Canada; Department of Anatomy and Cell Biology, Schulich School of Medicine and Dentistry, University of Western Ontario, London, ON, Canada; Department of Anatomy and Cell Biology, Schulich School of Medicine and Dentistry, University of Western Ontario, London, ON, Canada; Department of Anatomy and Cell Biology, Schulich School of Medicine and Dentistry, University of Western Ontario, London, ON, Canada

**Keywords:** auditory cortex, auditory processing, autism spectrum disorder, CASPR2, *Cntnap2*, evoked potentials, neurodevelopmental disorder, rat

## Abstract

The contactin-associated protein-like 2 gene, *CNTNAP2,* is a highly penetrant risk gene thought to play a role in the genetic etiology of language-related disorders, such as autism spectrum disorder and developmental language disorder. Despite its candidacy for influencing language development, few preclinical studies have examined the role of *CNTNAP2* in auditory processing. Using *in vivo* and *in vitro* electrophysiological recordings in a rat model with translational validity, we report that a loss of the *Cntnap2* gene function caused immature-like cortical evoked potentials, delayed multiunit response latencies to acoustic stimuli, impaired temporal processing, and led to a pattern of hyperexcitability in both multiunit and single cell recordings in adulthood. These collective results provide direct evidence that a constitutive loss of *Cntnap2* gene function in rats can cause auditory processing impairments similar to those seen in language-related human disorders, indicating that its contribution in maintaining cortical neuron excitability may underlie the cortical activity alterations observed in *Cntnap2*^−/−^ rats.

## Introduction

The genetic etiology of neurodevelopmental disorders involving altered speech and/or language ability is complex, with several genes thought to play a role (reviewed in Kang and Drayna 2011; Graham and Fisher 2015; Deriziotis and Fisher 2017). Over the past two decades, overlapping genetic pathways have been implicated in disorders where language-related phenotypes are a core defining feature, such as autism spectrum disorder (ASD) and developmental language disorder (DLD; Kang and Drayna 2011; Rodenas-Cuadrado et al. 2014; Poot 2015; Deriziotis and Fisher 2017). For example, the contactin-associated protein-like 2 (*CNTNAP2*) gene is a highly penetrant risk gene associated with both DLD and ASD (Arking et al. 2008; Vernes et al. 2008; Newbury et al. 2011). A homozygous loss of function in *CNTNAP2* causes cortical dysplasia, seizures, language impairment, and autistic features (Strauss et al. 2006; Rodenas-Cuadrado et al. 2016) with various heterozygous mutations leading to less severe phenotypes, typically including an impairment in speech and language, such as dysarthric language, language delay, or absent speech/language (reviewed in Rodenas-Cuadrado et al.  2014; Poot 2015). Moreover, genetic variation in *CNTNAP2* in neurotypicals has been shown to be responsible for differences in language processing and development (Whalley et al. 2011; Whitehouse et al. 2011; Worthey et al. 2013). Despite the clear links between *CNTNAP2* and language development, it remains unresolved whether differences in language processing, such as difficulties in processing the rapidly changing sounds associated with speech, arise due to *CNTNAP2*-mediated alterations in the response properties of neurons in the auditory cortex.

In general, neurophysiological studies on individuals with language-related disorders have reported differences in the processing of temporally manipulated sounds (Oram Cardy et al. 2004; Samson et al. 2011). Furthermore, sound-evoked cortical responses in individuals with ASD or DLD are often found to be delayed, reflected by slower response latencies (Gage et al. 2003; Roberts et al. 2010, 2019; Matsuzaki et al. 2012; Edgar et al. 2015; Berman et al. 2016; Port et al. 2016; cf. Madsen et al. 2015; reviewed in O’Connor 2012; Sinclair et al. 2016). As human maturation is typically associated with decreases in response latency (O’Connor 2012; Sinclair et al. 2016; cf. Ruhnau et al. 2011), the activation pattern observed in ASD and DLD is thought to reflect that of an “immature” cortex. Similar immature-like cortical profiles have been observed in some rodent models of ASD (Gandal et al. 2010; Rotschafer and Razak 2013; Engineer, Centanni, Im, Borland, et al. 2014a). Moreover, ASD-linked changes in the timing of neural activity in response to sensory stimuli (e.g., response onset and offset) have been suggested to result from altered cortical excitability (reviewed in Takarae and Sweeney 2017). In addition to differences in response latency, autism is associated with altered morphology of sound-evoked activity thought to originate from the primary auditory cortex, reflected by a larger temporal N1 (N1a) and a smaller frontal N1 (N1b) (Bruneau et al. 1999; Brandwein et al. 2015; reviewed in Sinclair et al. 2016). In the case of *CNTNAP2*-related deficits, it is possible that altered cortical response properties underlie the significant impairments in language; however, no studies have investigated whether a loss of function of this autism-linked gene causes delayed cortical responses to sounds and/or impaired ability for cortical neurons to accurately process rapidly presented sounds.

In this study, we used a *Cntnap2* knockout rat model, which has been shown to have considerable face validity for ASD-related behaviors (Scott et al. 2020), to assess if a loss of *Cntnap2* function results in immature auditory processing in adulthood reflected by changes in the temporal control of neural activity. More specifically, auditory temporal processing and cortical excitability were examined by recording neural population activity (i.e., evoked potentials and multiunit firing rates) from the auditory cortex of adult rats in response to temporally simple and complex acoustic stimuli using *in vivo* extracellular recordings with multichannel microelectrode arrays. Furthermore, as cortical excitability can result from an altered pyramidal neuronal function, we used whole-cell patch clamp recordings in the auditory cortex of *Cntnap2* knockout and wildtype rats to assess intrinsic neuronal properties and the resulting changes in the features and kinetics of generated action potentials, as well as postsynaptic responses. Finally, we investigated possible changes in the expression of markers for glutamatergic excitation and GABAergic inhibition as alterations in the inhibitory and excitatory systems have been hypothesized as a mechanism for altered cortical excitability in language-related disorders such as ASD and DLD, with the auditory cortex of autistic individuals having higher glutamate and lower GABA concentrations (Blatt and Fatemi 2011; Chattopadhyaya and Di Cristo 2012; Brown et al. 2013; Gaetz et al. 2014; Rojas et al. 2014). Overall, we show that, despite mature brainstem auditory-evoked potentials in adulthood, the cortical ability to process simple and complex temporally modulated sounds remains altered in adult *Cntnap2* knockout rats and is characterized by immature-like cortical evoked potentials, delayed multiunit latencies, impaired temporal processing, and a pattern of hyperexcitability in both multiunit and single-cell recordings.

## Materials and Methods

### Animals

Male Sprague–Dawley wildtype and homozygous knockout rats were used in this study. Homozygous knockout breeders were obtained from Horizon Discovery (Boyertown, PA; originally created at SAGE Laboratories, Inc. in conjunction with Autism Speaks; the line is now maintained by Envigo) and bred to obtain *Cntnap2*^−/−^ rats. The model contains a five base pair deletion in exon six of the *Cntnap2* gene, created using the zinc finger nuclease target site CAGCATTTCCGCACC|aatgga|GAGTTTGACTACCTG. Wildtype breeders from Charles River Laboratories (Wilmington, MA) were bred to obtain wildtype rats. Animals from a minimum of three litters of a given genotype were used in all experiments. Date of birth was designated as postnatal day zero (P0). Rats were weaned on P21, and sexes were separated on P35. Rats were housed in a temperature-controlled room on a 12 h light/dark cycle with ad libitum food and water. All procedures were approved by the University of Western Ontario Animal Care Committee and were in accordance with the guidelines established by the Canadian Council on Animal Care.

Prior to the collection of subdermal and *in vivo* extracellular electrophysiological recordings, all wildtype (*n* = 7) and *Cntnap2*^−/−^ (*n* = 8) rats were weighed and the differences in body mass analyzed, with no differences in body mass present (wildtype 503 ± 6 g, *Cntnap2*^−/−^ 500 ± 20 g; *P* = 0.88). Moreover, no genotypic differences existed in the age of the rats used (wildtype 88 ± 1 days, *Cntnap2*^−/−^ 92 ± 2 days; *P* = 0.09). Finally, no differences in hearing threshold existed between the genotype of rats used in the brainstem auditory-evoked potential (BAEP), cortical auditory-evoked potential (CAEP), and multiunit *in vivo* cortical recordings (wildtype 27.9 ± 1.0 dB SPL, *Cntnap2*^−/−^ 30.0 ± 0.9 dB SPL; *P* = 0.15). There were also no differences in the age of rats used for *in vitro* electrophysiological slice recordings (wildtype 91 ± 10 days, *Cntnap2*^−/−^ 87 ± 7 days; *P* = 0.77).

**Figure 1:**
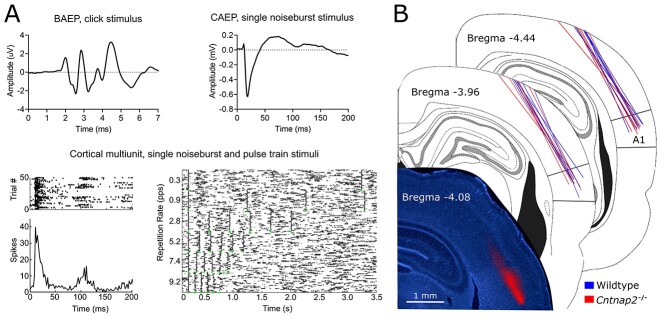
Responses to acoustic stimuli from central auditory areas in rats. (*A*) BAEP from a single rat elicited by the presentation of click stimuli (90 dB SPL); averaged CAEP from a representative multiunit cluster elicited by the presentation of a 50 ms noise burst stimuli (90 dB SPL); cortical spiking activity from a representative multiunit cluster elicited by the presentation of a 50 ms noise burst stimulus or from a six-pulse train composed of 25 ms noise bursts presented at six different repetition rates (90 dB SPL). Dot raster plot (dot = 1 spike; row = 1 trial) and/or line peristimulus time histogram (PSTH; 2 ms bins) shown. (*B*) Representative recording penetration in the primary auditory cortex (A1) in the rat, accompanied by schematics of the locations of electrode penetrations reconstructed from histological sections for each of the rats that underwent *in vivo* electrophysiological recordings (wildtype rats, *n* = 7 and Cntnap2^−/−^ rats, *n* = 8). Electrodes were advanced into the cortex falling between 3.72 and 4.56 mm caudal to bregma.

### Hearing Assessment and Collection of Brainstem Auditory Evoked Potentials

**Figure 2:**
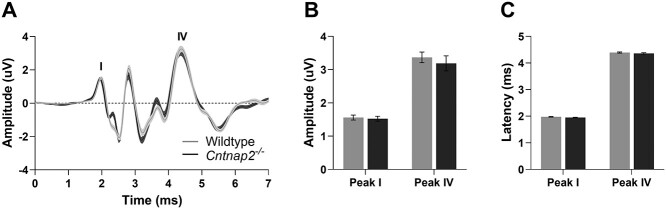
Intact auditory brainstem responses in adult *Cntnap2*^−/−^ rats. (*A*) Averaged BAEP waveforms from wildtype (*n* = 7) and *Cntnap2*^−/−^ rats (*n* = 8), in response to a 90 dB SPL, 0.1 ms click stimulus. Solid line and shaded region denote mean ± standard error (SE). (*B*) Amplitude and (*C*) latencies of peaks I and IV representing activity from the auditory nerve and lateral lemniscus terminating at the inferior colliculus respectively, represented as mean ± SE.

Hearing levels were assessed using BAEP (Petrova 2009), which was performed in a double-walled sound-attenuating chamber. Rats were anesthetized with ketamine (80 mg/kg; I.P.) and xylazine (5 mg/kg; I.P.), and subdermal electrodes (27 gauge; Rochester Electro-Medical, Lutz, FL) were positioned at the vertex, over the right mastoid process and on the midback in wildtype and homozygous knockout rats. The animal was not secured in a stereotaxic frame during the hearing assessment. Body temperature was maintained at approximately 37 °C using a homeothermic heating pad (507220F; Harvard Apparatus, Kent, UK). Sound stimuli were generated by a Tucker-Davis Technologies (TDT, Alachua, FL) RZ6 processing module at 100 kHz sampling rate and delivered by a magnetic speaker (MF1; TDT) positioned 10 cm from the animal’s right ear. The left ear was occluded with a custom foam earplug. Sound stimuli for the BAEP and electrophysiological recording experiments were calibrated with custom MATLAB software (The Mathworks, Natick, MA) using a 0.25 inch microphone (2530; Larson Davis, Depew, NY) and preamplifier (2221; Larson Davis). The auditory-evoked activity was collected using a low- impedance headstage (RA4L1; TDT), preamplified, and digitized (RA16SD Medusa preamp; TDT) and sent to a RZ6 processing module via a fiber optic cable. The signal was filtered (300–3000 Hz) and averaged using the BioSig software (version 5.5, TDT). Auditory stimuli consisted of a click (0.1 ms) stimulus that was presented 1000 times (21 times/s) at decreasing intensities from 90 to 20 dB sound pressure level (SPL) in 10 dB SPL steps. Near threshold, successive steps were decreased to 5 dB SPL, and each sound level was presented twice in order to best determine the BAEP threshold using the criteria of just noticeable deflection of the averaged electrical activity within the 10 ms window (Abitbol et al. 2016; Schormans et al. 2017; Scott et al. 2018). Evoked potentials collected in response to the 90 dB SPL stimulus were used for BAEP analysis (Figs 1*A* and 2). The peak amplitudes of each of waves I and IV were measured in microvolts in reference to the baseline (0 μV), and the latency of each of these peaks was determined from the stimulus onset. Data analyses were performed with the BioSig software (Tucker-Davis Technologies) and Microsoft Excel 2010 (Microsoft Corp.).

### *In Vivo* Electrophysiological Recordings

#### Surgical Procedure

Immediately following the BAEP measures, each rat was maintained under ketamine/xylazine anesthesia, the foam earplug was removed from the left ear, and the animal was fixed in a stereotaxic frame with blunt ear bars. Supplemental doses of ketamine/xylazine were administered IM as needed. A midline incision was made in the skin, and the underlying tissue was reflected from the skull. A headpost was fastened to the skull with dental acrylic, and a stainless steel screw was inserted into the right frontal bone to serve as an anchor for the headpost as well as electrical ground. A craniotomy (2.0 mm × 3.0 mm; 3.5–5.5 posterior to bregma and 0.0 to 3.0 medial the lateral ridge) was performed in the left parietal bone in order to expose the cortex. At the end of the surgical procedure, the right ear bar was removed to allow free-field auditory stimulation of the right ear during the electrophysiological recordings in the contralateral cortex. The rat remained securely positioned in the stereotaxic frame using the left ear bar and the headpost for the remainder of the experiment.

#### Recording Equipment

Extracellular electrophysiological signals were acquired using a 32-channel microelectrode array that consisted of a single 50-μm-thick shank with 32 equally spaced recording sites (50 μm apart), spanning 1.55 mm in length (A1x32-10 mm-50s-177-A32; NeuroNexus Technologies, Ann Arbor, MI). The electrode array was connected to a high-impedance headstage (NN32AC; TDT), and the neuronal activity was preamplified and digitized (two RA16SD Medusa preamps; TDT), and sent to a RZ5 processing module via a fiber optic cable. For each of the 32 channels, multiunit activity was digitally sampled at 25 kHz and band-pass filtered online at 3–300 Hz for local field potential (LFP) data and 300–3000 Hz for spiking data, using a voltage threshold for spike detection of 3 standard deviations (SDs) above the noise floor. All LFP activity and the timing of the detected spikes and their associated waveforms were stored for offline analyses.

#### Recording Sites

Two recording penetrations were conducted in each rat targeting the auditory cortex between 3.7 and 4.5 mm caudal to bregma. Using a high-precision stereotaxic manipulator (World Precision Instruments, Sarasota, FL), the electrode array was inserted in the cortex through a small slit in the dura using a dorsomedial-to-ventrolateral approach (30° angle), with the electrode array entering the cortex targeting first 4.0 mm caudal to bregma and 1.0 mm medial to the temporal ridge of the skull (i.e., ~4.6 mm lateral to midline). The second penetration targeted 4.3 mm caudal to bregma and 1.0 mm medial to the temporal ridge of the skull. For both penetrations, the electrode array was advanced at the 30° angle until all recording sites were within the cortex (depth of 1.55 mm) based on visual confirmation using a surgical microscope equipped with a high-resolution camera. A hydraulic microdrive (FHC; Bowdoinham, ME) was then used to slowly advance the electrode array into the auditory cortex (~4800 μm depth).

#### Acoustic Stimulation Paradigms

Overall, in each rat, acoustic stimulation paradigms were performed at two locations within the auditory cortex: 4.0 and 4.3 mm caudal to bregma. Before conducting electrophysiological recordings at each location, the electrode array was allowed to settle in place for 45 min. At each recording location, two auditory stimulation paradigms were presented using an RZ6 processing module (TDT; 100 kHz sampling rate) and custom MATLAB (The Mathworks) software. For both paradigms, acoustic stimuli consisted of broadband noise bursts (1–32 kHz) presented at 90 dB SPL from a magnetic speaker (MF1; TDT) positioned 10 cm above the surface of the stereotaxic frame and 10 cm from the base of the right pinna on a 30° angle from midline in the contralateral space. In the first paradigm, a 50 ms noise burst was presented every 3 to 5 s for a total of 50 presentations, which allowed for the quantification of the CAEP, and multiunit cluster cortical response dynamics. To determine the ability of the cortex to respond to rapidly presented stimuli (i.e., temporal response dynamics), the second paradigm consisted of a train of six discrete 25 ms noise bursts, with each six-pulse train presented 25 times at each of six repetition rates (0.3, 0.9, 2.8, 5.2, 7.4, 9.2 pulses per second [pps]). These repetition rates were chosen based on previous work that reported that the auditory cortex of wildtype rats begins to show a decrease in evoked activity to six pulses when the repetition rate exceeds ~9 pps (Kilgard and Merzenich 1998, 1999). Moreover, as it was predicted that the *Cntnap2*^−/−^ rats would show impaired temporal processing, these repetition rates were intended to reveal whether neurons in the auditory cortex of *Cntnap2*^−/−^ rats were unable to reliably fire action potentials in response to the final pulses of the faster rates (e.g., 7.4 and 9.2 pps), which would be evident as a decreased firing rate compared with the wildtype rats. The repetition rates were randomly interleaved to reduce adaptation effects, and a minimum of 2 s of silence separated each train.

#### Histological Confirmation of Penetrations

To allow for postexperiment histological reconstruction of the electrode penetrations, the electrode array was coated in DiI cell-labeling solution (V22885; Molecular Probes, Inc., Eugene, OR) prior to insertion into the cortex. At the completion of the electrophysiological experiment, the rat was injected with sodium pentobarbital (100 mg/kg; I.P.) in preparation for exsanguination via transcardial perfusion of 0.1 M phosphate buffer (PB; 300 mL) followed by 4% paraformaldehyde (400 mL). Next, the brain was removed and post-fixed in paraformaldehyde for 12 h, followed by storage in 30% sucrose/PB solution for cryoprotection. Using a microtome (HM 430/34; Thermo Fisher Scientific, Waltham, MA), frozen sections (40 μm) were cut in the coronal plane and collected serially. The sections were mounted in fluorescent DAPI mounting medium to label DNA (F6057 Fluoroshield with DAPI; Sigma-Aldrich, St. Louis, MO), and cover-slipped. Finally, fluorescent and brightfield images were obtained using an Axio Vert A1 inverted microscope (Carl Zeiss Microscopy GmbH, Jena, Germany) and ZEN lite imaging software (Carl Zeiss Microscopy). Each penetration was matched to its corresponding rostral-caudal location using the *Rat Brain in Stereotaxic Coordinates* (Paxinos and Watson 2007), and the penetrations were reconstructed (Fig. 1*B*). Penetrations that fell between 3.72 and 4.56 caudal to bregma were used for analysis.

#### Offline Analysis of Cortical Auditory Evoked Potentials

**Figure 3:**
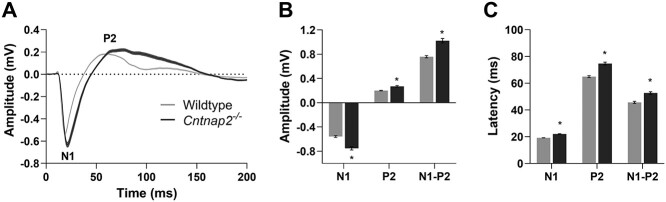
*Cntnap2*  ^−/−^ cortical auditory evoked potentials in adulthood reflect an immature-like profile. (*A*) Averaged CAEP waveforms from wildtype (*n* = 180 waveforms) and *Cntnap2*^−/−^ (*n* = 161 waveforms) rats in response to a 90 dB SPL noise burst. Solid line and shaded region denote mean ± SE. (*B*) Increased amplitudes and (*C*) prolonged latencies of the N1 and P2 potentials reflect an immature-like response profile in the adult rats. Data represented as mean ± SE. ^*^P < 0.05.

CAEPs were analyzed following the presentation of the 50 ms noise burst stimulus, in order to assess sound-evoked activity. For each penetration, auditory-evoked LFP activity was examined within 500 ms of the onset of the noise burst using custom MATLAB scripts (R2020a; The MathWorks). For each channel and recording location, mean LFPs were calculated by averaging the LFP recordings across all 50 trials. Using the mean LFP, the N1 and P2 amplitudes and latencies were determined, as these are well-established response measures of LFP recordings (Kurt et al. 2008; Eggermont et al. 2011; Engineer, Centanni, Im, Borland, et al. 2014a; Nagy et al. 2015). The most negative peak occurring within 40 ms of the noise burst stimulus was taken as the N1 peak. The most positive peak occurring after the N1 peak and within 100 ms of the noise burst stimulus was taken as the P2 peak. Finally, to create a compound CAEP trace and average amplitude and latency measures for each genotype, mean LFPs as well as the corresponding amplitude and latency were averaged across all channels (Fig. 3). Data analyses were performed with MATLAB (R2020a; The MathWorks).

#### Offline Analysis of Multiunit Activity

**Figure 4:**
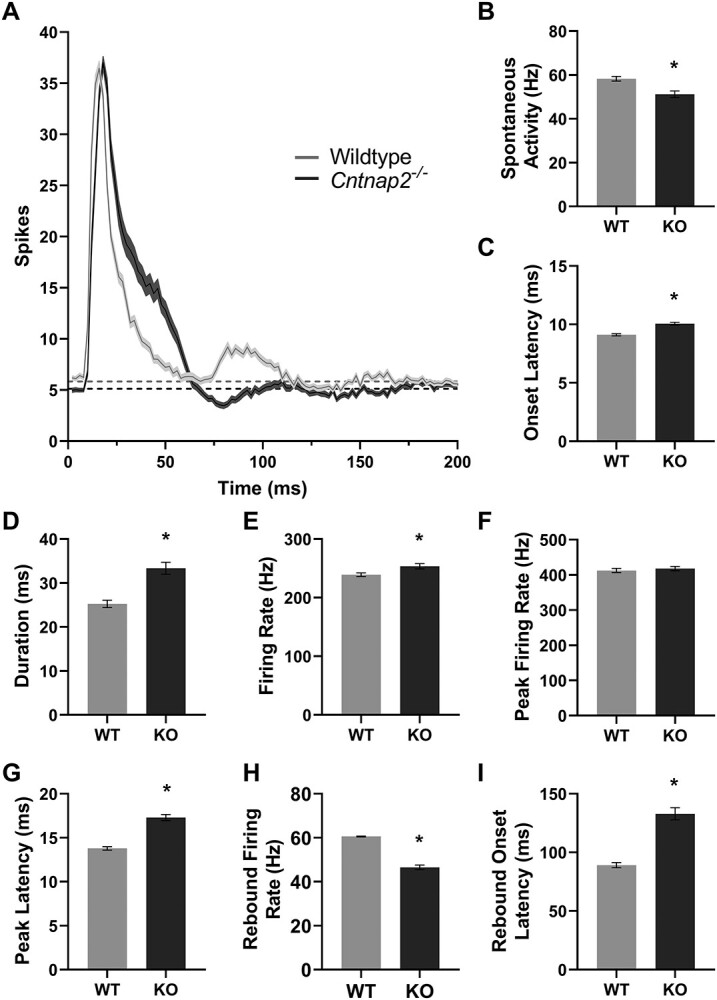
Spiking profiles in *Cntnap2*^−/−^ rat auditory cortex is hyper-responsive. (*A*) Multiunit cortical response dynamics in the primary auditory cortex to a 50 ms noise burst stimulus (90 dB SPL), represented as an averaged line PSTH from wildtype (*n* = 180 clusters) and *Cntnap2*^−/−^ (*n* = 161 clusters) rats. Solid line and shaded region denote mean ± SE. Horizontal dashed line represents spontaneous activity. (*B*) Spontaneous activity, (*C*) response onset latency, (*D*) response duration, (*E*) average response firing rate, (*F*) peak firing rate, (*G*) peak latency, (*H*) the rebound response firing rate, and (*I*) the onset of the rebound response represented as mean ± SE. Longer response duration and increased firing rate are indicative of a hyperexcitable response. ^*^P <0.05.

Auditory responsiveness was examined for each multiunit cluster in response to a single 50 ms noise burst. To do so, rasters and peristimulus time histograms (PSTHs) were generated for each multiunit cluster. Spontaneous activity was calculated by tallying the number of spikes within the last 500 ms of each trial, and then calculating the average spontaneous firing rate across all 50 trials (Hz/trial; Schormans et al. 2017). Prior to examining the response dynamics of each genotype, spiking activity of each multiunit cluster was measured within a 35 ms time window that was time-locked to 5–40 ms from stimulus onset, which ultimately allowed us to determine whether a multiunit cluster was responsive to acoustic stimulation. A multiunit cluster was considered to be responsive to acoustic stimulation if the firing rate within the 35 ms time window was significantly increased compared with the spontaneous activity as determined with a paired *t*-test (α = 0.05). Nonresponsive multiunits were removed from further analysis. To accurately assess multiunit latency differences between the two genotypes, spiking activity within a 500 ms time window after stimulus onset was parsed into 2 ms bins (Fig. 1*A*). Onset latency was defined as the first time the firing rate within a 2 ms bin surpassed 3 SDs above the spontaneous firing rate (Engineer, Centanni, Im, Rahebi, et al. 2014b; Tao et al. 2016). Offset latency was defined as the time at which the firing rate returned to the spontaneous firing rate for two consecutive bins (i.e., 4 ms). Response duration was then calculated as the time between the response onset latency and response offset latency, and defined as the response window. The response magnitude of each multiunit was then determined by tallying the number of spikes within the response window for each trial, and dividing by the response duration, to determine the average firing across the 50 trials (Hz/trial). The peak firing rate and latency were measured by determining the maximum firing rate within a 2 ms bin that was located within the response window, and the time at which it occurred. Finally, to further examine the temporal response features of these multiunit clusters to simple acoustic stimulation, the post response period (i.e., the spiking activity following the presentation of the acoustic stimulus) was quantified in each of the genotypes. More specifically, the onset and offset latency as well as the firing rate were calculated for the rebound response (i.e., the increased neural activity observed following a period of suppression; see Eggermont 1991). The onset latency of the rebound response was defined as the first time the firing rate within a 2 ms bin was above the spontaneous firing rate for 8 ms. To calculate the firing rate for the rebound response, the offset latency (i.e., the time at which the firing rate returned to the spontaneous firing rate for 8 ms) and the onset latency were used to calculate the duration of the rebound response. The magnitude of the rebound response was then determined by counting the number of spikes within the duration of the response for each trial, and then dividing by the duration, to determine the average firing rate across the 50 trials (Hz/trial). All described metrics were averaged across multiunit clusters for each genotype (Fig. 4).

**Figure 5:**
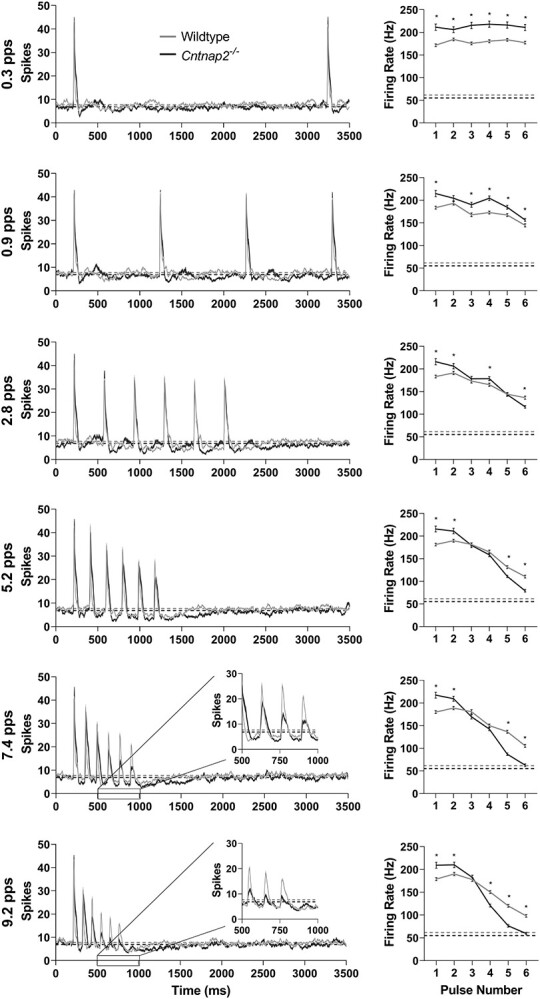
*Cntnap2*  ^−/−^ rats have a reduced ability to consistently respond to a six-pulse noise burst train. (Left) responses of multiunit clusters in the primary auditory cortex to a six-pulse train of 25 ms noise burst stimuli (90 dB SPL), presented at repetition rates of 0.3, 0.9, 2.8, 5.2, 7.4, and 9.2 pps, represented as an averaged line PSTH from wildtype (*n* = 176 clusters) and *Cntnap2*^−/−^ (*n* = 161 clusters) rats. Solid line and shaded region denote mean ± SE. Horizontal dashed line represents spontaneous activity. (Right) average firing rate in a 35 ms window (5–40 ms after stimulus onset) in response to each noise burst in the six-pulse train for each repetition rate, represented as mean ± SE. Horizontal dashed line represents spontaneous activity. As the repetition rate increases, the ability of multiunit clusters to respond to each noise burst stimulus in the six-pulse train decreases; this effect is more pronounced in the responses of *Cntnap2*^−/−^ rats at rates of 5.2 pps and greater. ^*^P <0.05.

Auditory temporal processing was examined for each multiunit cluster in response to 25 ms noise bursts presented at six different repetition rates. Similar to the analysis described above, spontaneous activity was calculated within the final 2 s of each trial and averaged across all trials. The responsivity of each multiunit cluster was determined for each noise burst in the six-pulse train by calculating the spiking activity in a 35 ms response window, which was time-locked to 5–40 ms from each stimulus onset (Kilgard et al. 2001; Schormans et al. 2017). For a multiunit cluster to be considered responsive to a given pulse, it needed to show a significantly increased firing rate compared with the spontaneous activity as determined with a paired *t*-test (α = 0.05). Multiunits without an acoustic response to the first pulse were removed from analysis. Unlike the auditory responsiveness analysis, spiking activity of each multiunit in response to the six different repetition rates was parsed into 5 ms bins (Bao et al. 2004; Zhou and Merzenich 2008) for both the raster and PSTH plots (Fig. 5). To determine the effects of increasing repetition rate as well as to allow the comparison of cross-sites and genotypes, the repetition rate transfer function (RRTF) was determined (Kilgard and Merzenich 1998, 1999). The response magnitude evoked by the last five pulses in the train (pulses 2–6) was averaged for each of the repetition rates and then compared between genotypes. To account for differences in response magnitude between the wildtype and *Cntnap2*^−/−^ rats in response to the first pulse as well as variability in the number of neurons included in different multiunit responses, the mean temporal modulation-transfer functions (tMTFs) were calculated as it provides the normalized cortical response as a function of the repetition rate (see equation (1); Bao et al. 2004; Chang et al. 2005; Zhou and Merzenich 2008).

(1) \begin{equation*} \mathrm{tMTFs}=\frac{\mathrm{avg}\left({\mathrm{FR}}_{2-6}\right)}{{\mathrm{FR}}_1} \end{equation*}

**Figure 6:**
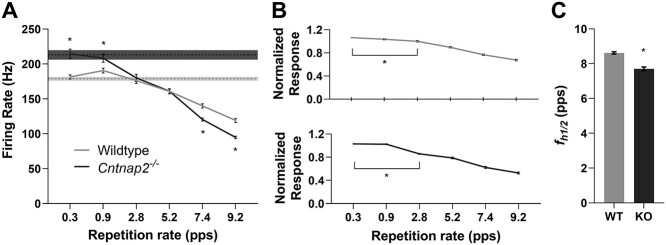
Poor temporal processing is reflected in the decreased rate-following ability in *Cntnap2*^−/−^ rats. (*A*) RRTF reveals that the subsequent capacity of *Cntnap2*^−/−^ multiunit clusters is poorer than wildtypes at repetition rates of 7.4 and 9.2 pps despite knockout clusters having an increased firing rate as compared with the first noise burst in the six-pulse train. The average firing rate to noise bursts 2–6 in the six pulse train are represented by the solid line, with error bars denoting SE. Dashed line and shaded region represent the mean firing rate ± SE to the first noise burst. (*B*) The tMTF normalizes the RRTF to the firing rate in response to the first noise burst stimulus showing that in both wildtype and *Cntnap2*^−/−^ rats, the ability of multiunit clusters to follow repeated stimuli begins to decrease at a repetition rate of 2.8 pps. (*C*) The *f_h1/2_* confirms that *Cntnap2*^−/−^ multiunit clusters have a poorer capacity for processing high-rate stimuli as the rate at which the tMTF of each cluster was at half its maximum is lower in *Cntnap2*^−/−^ rats compared with wildtypes. Data represented as mean ± SE. ^*^P < 0.05.

**Figure 7:**
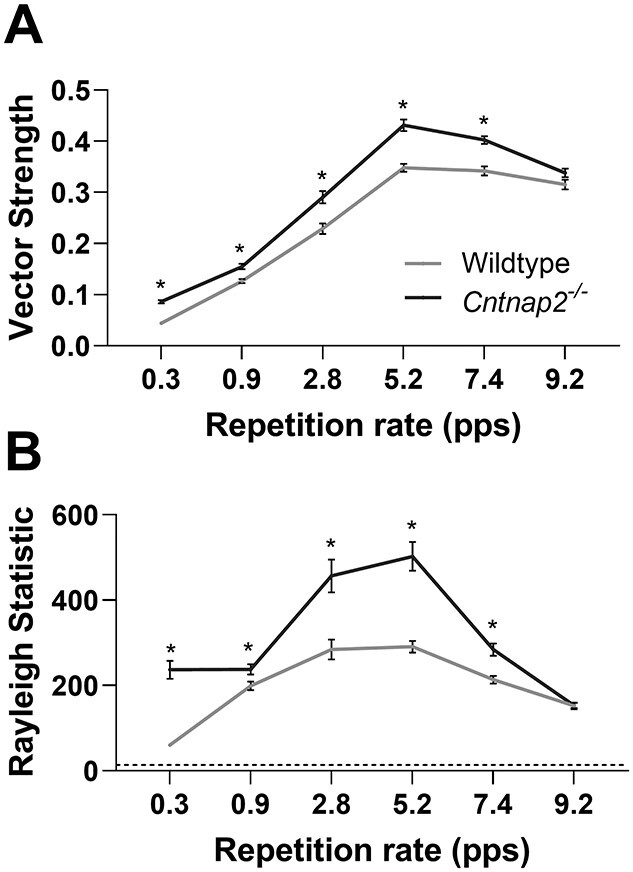
No deficit in the cortical phase-locking of multi-unit responses in *Cntnap2*^−/−^ rats. (*A*) Cortical spike timing in relation to stimulus phase was determined using vector strength, measured at the various repetition rates for each multi-unit cluster. A higher value signifies more precise spike timing. (*B*) The significance of the phase locking is measured with the Rayleigh statistic, where >13.82 indicates *P* < 0.001. Data represented as mean ± SE. ^*^P < 0.05.

More specifically, for each repetition rate, the average response magnitude evoked by the last five noise bursts in the six-pulse train (pulses 2–6) was divided by the response magnitude evoked by the first noise burst in the train. The ability of the cortex to follow the rapidly presented stimuli was then estimated for each multiunit by interpolating the highest temporal rate at which the tMTF was at least half of its maximum, referred to as *f*_h1/2_ (Fig. 6; Zhou and Merzenich 2008). For multiunits that did not reach half maximum at the highest temporal rate, the *f*_h1/2_ was defined as the highest temporal rate (i.e., 9.2 pps). Finally, temporal fidelity, or the capacity of cortical neurons to respond in a time-locked fashion to each pulse at each repetition rate, was quantified using vector strength and Rayleigh statistic, taking into account the total number of spikes generated in the full phase of each pulse (i.e., all spikes in response to a given noise burst up to the onset of the following noise burst; Bao et al. 2004; Chang et al. 2005; Zhou and Merzenich 2008). Vector strength and the Rayleigh statistic were calculated using the toolbox for circular statistics in MATLAB (Berens 2009). All described metrics were averaged across multiunit clusters for each genotype (Fig. 7). Data analyses were performed with MATLAB (R2020a; The MathWorks).

### In Vitro Electrophysiological Recordings Slice Preparation

Sprague–Dawley wildtype (*n* = 6) and homozygous knockout (*Cntnap2*^−/−^*; n* = 4) rats were anesthetized with isofluorane and their brains quickly removed and transferred into ice-cold slicing solution containing (in mM): 2.5 KCl, 1.25 NaH_2_PO_4_-H_2_O, 24 NaHCO_3_, 10 MgSO_4_, 11 glucose, 234 sucrose, 2 CaCl_2_, 3 myoinositol, 2 Na-pyruvate, and 0.4 ascorbate; equilibrated with 95% O_2_/5% CO_2_. Coronal slices (3.7 and 4.5 mm caudal to bregma) of 300 μm thickness were cut with a vibrating microtome (Compressotome VF-200) in a chamber filled with ice-cold preparation solution and then transferred into a holding chamber filled with artificial cerebrospinal fluid (ACSF) containing (in mM): 3 KCl, 1.25 NaH_2_PO_4_-H_2_O, 3 MgSO_4_, 26 NaHCO_3_, 124 NaCl, and 10 glucose; equilibrated with 95% O_2_/5% CO_2_. CaCl_2_ (2 mM) was added to the ACSF a few minutes before slices were transferred. ACSF was heated to approximately 35 °C for 30 min in order to improve patching success, and the slices were left to rest for an additional 1 h at room temperature to recover. Slices were kept at room temperature during the experiment.

#### Whole-Cell Recordings

Electrophysiological experiments were performed as reported previously (Bosch and Schmid 2006; Simons-Weidenmaier et al. 2006; Zaman et al. 2017). Pyramidal cells from the auditory cortex layers 2/3 were visualized through an upright microscope (Zeiss Axioskop, Germany), equipped with an EMCCD camera (Evolve 512, Photometric, Tuscon, AZ). Recording electrodes were pulled on a P-97 Puller (Sutter Instrument, Novato, CA) from fabricated borosilicate glass capillaries (1B150F-4, OD: 1.50 mm, ID: 0.84 mm, World Precision Instruments, Sarasota, FL) and had 3–7 ᴍΩ resistance when filled with an intracellular solution containing the following (in mM): 140 K-gluconate, 10 KCl, 1 MgCl2, 10 HEPES, 0.02 EGTA, 3 Mg-ATP, and 0.5 Na-GTP, pH adjusted to 7.3, 290–300 mosm/L. Signals were sampled at 5 kHz, amplified with Axopatch 200B, digitized with Digidata-1550, and analyzed using pClamp10.4 (all Axon Instruments, Molecular Devices, Sunnydale, CA). Only pyramidal cells with access resistance <25 ᴍΩ were included in analyses, and parameters were monitored throughout recordings.

**Figure 8:**
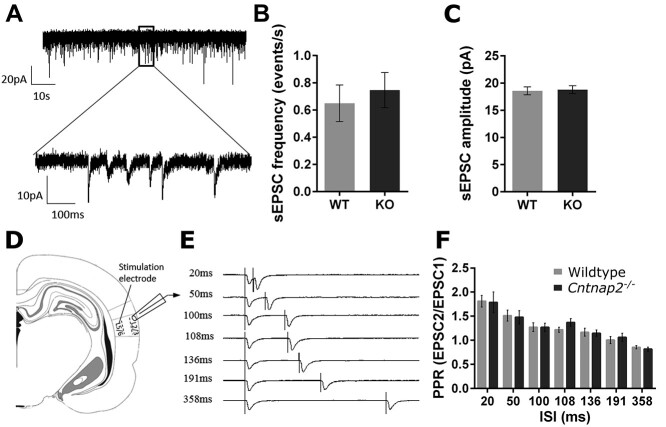
*Cntnap2*  ^−/−^ neurons are synaptically comparable to wildtype neurons. (*A*) Sample recording trace of spontaneous EPSCs from the auditory cortex of an adult wildtype rat with the (*B*) mean frequency and (*C*) mean amplitudes. (*D*) Representative recording areas including the primary auditory cortex (A1) in the rat, accompanied by the positions of the recording and stimulating electrodes in layers 2/3 and layers 5/6, respectively. (*E*) Sample traces of paired pulse evoked EPSCs from a wildtype rat at various ISIs, vertical lines represent stimulation pulses, and (*F*) paired-pulse ratios (amplitude of the second EPSC divided by first EPSC). All data are represented as mean ± SE (*B*, *C*: wildtype *n* = 19 cells, *Cntnap2*^−/−^  *n* = 13 cells; *F*: wildtype *n* = 12 cells, *Cntnap2*^−/−^  *n* = 6 cells). ^*^P <0.05.

**Figure 9:**
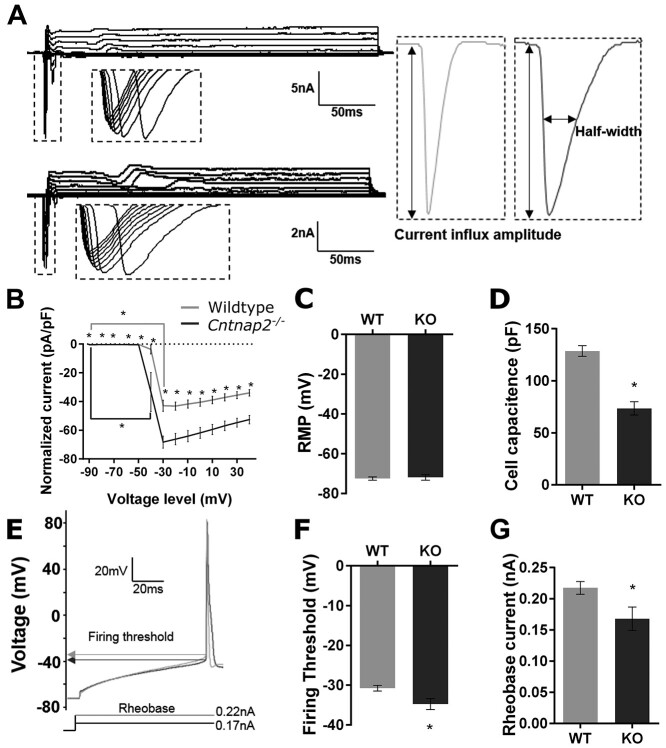
*Cntnap2*  ^−/−^ neurons are generally more excitable. (*A*) Sample voltage-clamp recordings from wildtype (top left) and *Cntnap2*^−/−^ (bottom left) neurons and representative magnified fast inward currents from wildtype (grey) and *Cntnap2*^−/−^ (black) voltage-clamp recordings. (*B*) No differences in resting membrane potential, but (*C*) *Cntnap2*^−/−^ neurons have a lower cell capacitance. (*D*) Voltage-clamp recordings normalized to cell capacitance reveal *Cntnap2*^−/−^ neurons have a higher current density of transient voltage-activated inward currents and activation at lower voltages. (*E*) Representative current-clamp recording of action potentials from wildtype (grey) and *Cntnap2*^−/−^ (black) neurons with firing threshold and rheobase indicated. (*F*) The action potential firing threshold and (*G*) rheobase current at which the neurons fire the first action potential reveal *Cntnap2*^−/−^neurons are more excitable. All data are represented as mean ± SE (*B*: wildtype *n* = 16 cells, *C*, *D*: wildtype *n* = 17 cells, *F*: wildtype *n* = 15 cells, *G*: wildtype *n* = 16 cells; *B*–*G*: *Cntnap2*^−/−^  *n* = 10 cells). ^*^P <0.05.

Whole-cell voltage clamp electrophysiology of pyramidal neurons in layers 2/3 of A1 (wildtype: *n* = 6 rats, 14–16 cells; *Cntnap2*^−/−^: *n* = 4 rats, 8–13 cells) was conducted to assess the spontaneous and evoked EPSC activity, as well as cell capacitance and membrane resistance. The membrane potential was held at −70 mV for all voltage-clamp recordings. sEPSCs were assessed by 5 min recordings of cell currents. For the evoked EPSCs, layers 5/6 of A1 were stimulated using a bipolar tungsten electrode (Science Products), and paired pulses were generated by a pulse generator (Master-8, AMPI, Israel) in wildtype and *Cntnap2*^−/−^ cells. To determine paired-pulse ratios, interstimulus intervals (ISIs) of 20, 50, 100, 108, 136, 191, and 358 ms were used (Fig. 8). Voltage clamp step recordings were measured by holding the membrane potential at −70 mV and hyper- or depolarizing the membrane for 300 ms to −90 mV to +40 mV in 10 mV increments (Fig. 9).

**Figure 10:**
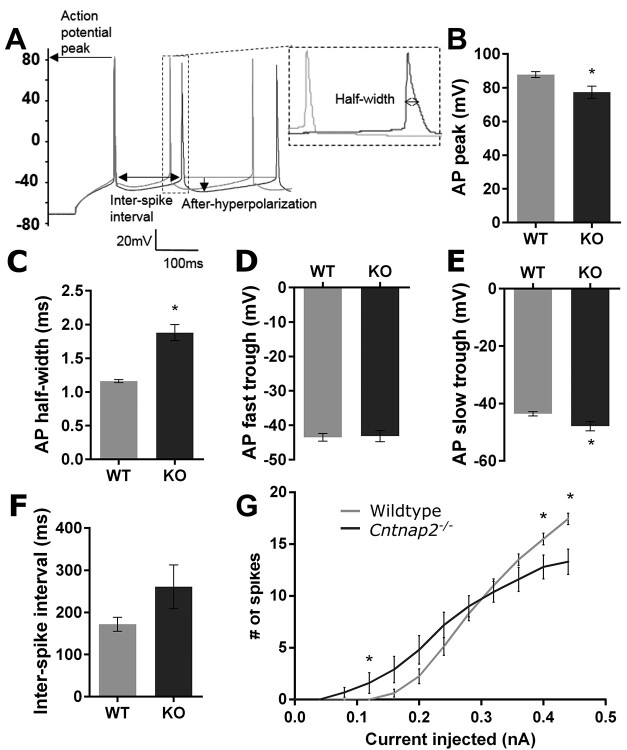
*Cntnap2*  ^−/−^ neurons have delayed currents and fire more easily but elicit fewer maximum spikes. (*A*) Sample current clamp recordings of wildtype (grey) and *Cntnap2*^−/−^ (black) neurons with labeled metrics of interest. Action potential features presented are (*B*) action potential peak voltage, (*C*) action potential half-width, (*D*) fast trough of after-hyperpolarization, (*E*) after-hyperpolarization slow trough, and (*F*) first interspike interval. (*G*) Firing frequency in response to increasing steady-state current injections as indicated. All data are represented as mean ± SE (*B*, *G*: wildtype *n* = 16 cells; *C*, *F*: wildtype *n* = 15 cells; *D*: wildtype *n* = 17 cells; *E*: wildtype *n* = 14 cells; *B*–*G*: *Cntnap2*^−/−^*n* = 10 cells). ^*^P <0.05.

Current-clamp was used to assess resting membrane potentials in wildtype (*n* = 6 rats, 14–17 cells) and *Cntnap2*^−/−^ rats (*n* = 4 rats, 10 cells). Current clamp recordings were made at resting membrane potentials of the cells and involved 1 s long step current injections in 40 pA increments from −120 pA to +480 pA. These recordings were used to assess firing threshold, rheobase, action potential features and kinetics, interspike intervals, and firing rates (Fig. 10).

#### Offline Analysis

The frequencies and amplitudes of sEPSCs were analyzed in MiniAnalysis (Synaptosoft, Fort Lee, NJ, USA). Voltage clamp step recordings, eEPSCs, and all current clamp recordings were analyzed in pClamp (Molecular Devices). The amplitudes of eEPSCs were taken from the EPSC generated by the first pulse across all the ISIs presented. The paired pulse ratio was calculated as the amplitude of the second eEPSC divided by the first eEPSC. Action potential half-width was measured as the width (ms) of an action potential at half its amplitude (firing threshold peak). Action potential after hyperpolarization was examined for the first action potential. The fast trough was measured as the difference between the AP baseline and trough within 5 ms following the action potential whereas slow trough was the difference between the baseline and the lowest trough between two successive action potentials. Interspike interval was the time from the baseline of the first action potential to the baseline of the second action potential. Action potential threshold was the baseline voltage of the first action potential, and the rheobase was the accompanying current that elicited the first action potential. For the firing rates, the number of spikes during the 1 s of step current stimulation was counted. Data analyses were performed with pClamp10.4 (Molecular Devices), MiniAnalysis software (Synaptosoft, Fort Lee, NJ, USA), and/or Microsoft Excel 2010 (Microsoft Corp.).

#### Data Presentation and Statistics

Graphs were generated with GraphPad (Prism 8.3.0 for Windows, GraphPad Software, San Diego, CA). Statistical analyses were conducted using IBM SPSS Statistics for Windows, Version 26.0 (IBM Corp., Armonk, NY). Statistical tests performed were based on the experimental design and included independent sample *t*-tests, one-way analysis of variance (ANOVA), and two-way, or three-way repeated measures analysis of variance (RM-ANOVA). In cases where independent sample *t*-tests were performed, Levene’s test was used to assess the equality of variances. In cases where a RM-ANOVA was performed, the Mauchly test was used to report a violation of the assumption of sphericity, such that the degrees of freedom were corrected using the Greenhouse Geisser (if ɛ < 0.75) or the Huynh–Feldt method (if ɛ > 0.75). Post-hoc one-way ANOVAs and paired-sample *t*-tests with a Dunnet’s or Bonferroni-corrected significance levels were used to compare differences in the group means in the case of a significant interaction. Differences were considered statistically significant when *P*-values (adjusted) were smaller than α = 0.05. Exact *P* values are reported, except in cases where *P* <0.001. Next we provide a summary of the various statistical tests performed in each of the sections. For complete statistical reporting, see Appendix B: Statistics Table 1.

#### Animal Characteristics

To compare differences in the body mass, age, and hearing threshold between wildtype and *Cntnap2*^−/−^ rats, independent sample *t*-tests were performed.

#### BAEP, CAEP, Multiunit Response Dynamics to a Single Noise Burst

To determine the effect of genotype on brainstem auditory-evoked potential amplitude and latency, a two-way RM-ANOVA was performed for peak (I, IV) × genotype (wildtype, *Cntnap2*^−/−^). Independent sample *t*-tests were used to compare the amplitude and latency of the N1 and P2 components of the cortical auditory-evoked potential, and subsequent derived metrics, between wildtype and knockout rats. Furthermore, in addition to comparing the spontaneous activity between the *Cntnap2*^−/−^ and wildtype rats, independent sample *t*-tests were also used to assess any genotypic differences in the response features of multiunit cluster spiking activity to a single 50 ms noise burst, including the response onset, response duration, average response firing rate, peak firing rate, latency to the peak firing, rebound response firing rate, and rebound onset latency.

#### Multiunit Temporal Processing and Temporal Fidelity

To assess whether auditory temporal processing ability differed between wildtype and *Cntnap2*^−/−^ rats, the multiunit firing rate (i.e. within in a 35 ms window) in response to each pulse in a six-pulse train presented at six different repetition rates was compared using a three-way RM-ANOVA, performed for pulse number (1, 2, 3, 4, 5, 6) × repetition rate (0.3, 0.9, 2.8, 5.2, 7.4, 9.2 pps) × genotype (wildtype, *Cntnap2*^−/−^). As a significant three-way interaction was found, post-hoc Bonferroni-corrected *t*-tests were performed to assess the presence of genotypic differences at each pulse. A two-way RM-ANOVA was performed on the RRTF for repetition rate (0.3, 0.9, 2.8, 5.2, 7.4, 9.2 pps) × genotype (wildtype, *Cntnap2*^−/−^) to further quantify differences in temporal processing and followed up with post-hoc Bonferroni-corrected *t*-tests for genotype. To determine the pulse rate at which a given genotype’s cortical response began to degrade, a one-way ANOVA for repetition rate (0.3, 0.9, 2.8, 5.2, 7.4, 9.2 pps) was performed on each genotype, and followed by post-hoc Dunnet’s *t*-test comparing each rate to 0.3 pps. Finally, genotypic differences in the capacity of cortical neurons to process high-rate stimuli was determined using an independent sample *t*-test on *f_h1/2_*. Using a two-way repeated measures ANOVA for repetition rate (0.3, 0.9, 2.8, 5.2, 7.4, 9.2 pps) × genotype (wildtype, *Cntnap2*^−/−^), vector strength and Rayleigh statistics were assessed to compare the temporal fidelity of wildtype and knockout multiunit clusters. As a significant interaction was found, post-hoc Bonferroni-corrected *t*-tests were completed for genotype.

#### Whole-Cell Response Properties

Genotypic differences in pyramidal cell function was assessed using a series of independent sample *t*-tests for cell properties including sEPSC frequency and amplitude, resting membrane potential, cell capacitance, spike firing threshold, rheobase current, and action potential features comprising of peak amplitude, half-width, fast trough, slow trough, and interspike interval. The paired-pulse ratios were analyzed using a two-way RM-ANOVA for ISI (20, 50, 100, 108, 136, 191, 358 ms) × genotype (wildtype, *Cntnap2*^−/−^). A two-way RM-ANOVA for voltage holding level (−90, −80, −70, −60, −50, −40, −30, −20, −10, 0, 10, 20, 30, 40 mV) × genotype (wildtype, *Cntnap2*^−/−^) followed by post-hoc Bonferroni-corrected *t*-tests were competed for genotype to assess differences in the rapid influx currents between wildtype and *Cntnap2*^−/−^ cells. Next, to determine the voltage required to elicit a current influx, a one-way ANOVA for voltage holding level (−90, −80, −70, −60, −50, −40, −30, −20, −10, 0, 10, 20, 30, 40 mV) within each genotype was performed followed by post-hoc Dunnet’s *t*-tests comparing each voltage holding level to −90 mV. The influence of genotype on the relationship between current injection level and number of spikes released was assessed using a two-way RM-ANOVA for current injection level (0.08, 0.12, 0.16, 0.20, 0.24, 0.28, 0.32, 0.36, 0.40, 0.44 nA) × genotype (wildtype, *Cntnap2*^−/−^) followed by post-hoc Bonferroni-corrected *t*-test for genotype.

## Results

### Functional Loss of *Cntnap2* Leads to a Larger and Delayed Auditory-Evoked Potential in the Cortex, but not the Brainstem, of Adult Rats

To determine whether a functional loss of *Cntnap2* causes differential changes in auditory-evoked responses at various levels throughout the central auditory pathway, electrophysiological recordings were performed in the auditory brainstem and cortex of wildtype (*n* = 7) and *Cntnap2*^−/−^ rats (*n* = 8, Fig. 1). BAEPs in response to a click stimulus were recorded using subdermal electrodes positioned on the scalp whereas CAEPs were recorded later in response to a noise burst (50 ms) using a microelectrode array inserted into the auditory cortex. At the level of the brainstem, there were no genotypic differences in the amplitude of the first or fourth peaks of BAEP (representative of activity in the auditory nerve and lateral lemniscus/inferior colliculus, respectively, Fig. 2). In contrast, both the N1 and P2 components of the CAEP were significantly larger in *Cntnap2*^−/−^ rats (*P* <0.001), resulting in a 35% increase in the peak-to-peak N1–P2 potential amplitude compared with the wildtypes (*P* <0.001; Fig. 3). Furthermore, there was a differential effect observed in the latency of the evoked responses such that cortical activity in the *Cntnap2*^−/−^ rats had a delayed speed of transmission of the N1 (*P* <0.001) and P2 (*P* <0.001) potentials and prolonged N1–P2 interpeak latency (*P* <0.001; Fig. 3*C*) whereas there were no genotypic differences in response latency in the brainstem nuclei (Fig. 2*C*). Taken together, these findings establish that *Cntnap2*-related alterations in auditory-evoked potentials manifest as both a larger and delayed response at the level of the auditory cortex, but not the brainstem, of adult rats.

### Decreased Spontaneous Activity in the Auditory Cortex of *Cntnap2*  ^−/−^ Rats

Prior to examining the consequences of a loss of *Cntnap2* on the response features of neurons within the auditory cortex (Fig. 4), the spontaneous multiunit activity was quantified in wildtype and *Cntnap2*^−/−^ rats. For each multiunit cluster (wildtype *n* = 180; *Cntnap2*^−/−^  *n* = 161), spontaneous activity was calculated within the last 500 ms window of each trial and then averaged across trials (mean spontaneous activity is denoted by the dashed horizontal line in Fig. 4*A*). Overall, there was a genotypic difference in spontaneous activity, such that the *Cntnap2*^−/−^ rats had lower spontaneous firing rates compared with wildtype rats (*P* <0.001; Fig. 4*B*).

### Multiunit Activity in the Auditory Cortex of *Cntnap2*  ^−/−^ Rats Is Hyper-responsive to Sound

To investigate the effect of a functional loss of *Cntnap2* on the response dynamics of neurons within the auditory cortex (Fig. 1), the spiking activity of multiunit clusters (wildtype *n* = 180; *Cntnap2*^−/−^  *n* = 161) were recorded following acoustic stimulation with single noise bursts (50 ms). As seen in the group averaged line peristimulus time histograms derived from the spiking responses to the noise burst stimulation (Fig. 4*A*), there was a genotypic difference in several response features, including a delay in both the response onset (*P* <0.001; Fig. 4*C*) and peak (*P* <0.001; Fig. 4*F*), as well as a longer response duration (*P* <0.001; Fig. 4*D*) and increased response magnitude (*P* = 0.01; Fig. 4*E*) in *Cntnap2*^−/−^ rats compared with the wildtypes. Taken together, these findings are indicative of a prolonged, hyper-responsiveness to acoustic stimulation of neurons in the auditory cortex of rats with a constitutive loss of *Cntnap2* function. In addition to quantifying the response dynamics of the initial response to single noise bursts, we also examined the rebound response of multiunit clusters, which typically occurs around 90 ms in wildtype rats. In contrast to the initial response to acoustic stimulation, the firing rate of the rebound response was reduced (*P* <0.001; Fig. 4*H*) and the onset of this response was delayed (*P* <0.001; Fig. 4*I*) in the *Cntnap2*^−/−^ rats compared with wildtype rats.

### Poorer Temporal Processing and Altered Temporal Fidelity in the Auditory Cortex of *Cntnap2*  ^−/−^ Rats

To further assess how auditory temporal processing was affected by a loss of *Cntnap2* function, the spiking activity of the multiunit clusters recorded from auditory cortex of wildtype (*n* = 176 clusters) and *Cntnap2*^−/−^ rats (*n* = 161 clusters) were compared in response to pulse trains consisting of six noise bursts (25 ms) presented at different repetition rates (0.3, 0.9, 2.8, 5.2, 7.4, and 9.2 pps; Fig. 1). Overall, a differential effect was evident in the spike firing rates (i.e., responsivity) between the genotypes across the various pulse repetition rates (i.e., significant interaction of repetition rate × pulse number × genotype: *P* <0.001; Fig. 5). Most notably, close inspection of the spike firing rates to the sixth pulse of the various trains shows that although *Cntnap2*^−/−^ multiunit clusters had a greater spiking activity than the wildtype multiunit clusters at the low pulse repetition rates (e.g., 0.3 pps: *P*_Bonf_ <0.001), this pattern was reversed at the faster pulse repetition rates with the knockout multiunit clusters showing significantly lower spike firing rates to the sixth pulse of the 5.2 pps (*P*_Bonf_ <0.001), 7.4 pps (*P*_Bonf_ <0.001) and 9.2 pps trains (*P*_Bonf_ <0.001), indicating that cortical neurons in knockout rats have difficulties following rapidly presented acoustic signals.

In an effort to further quantify this genotypic difference in auditory temporal processing, an RRTF was calculated in which the average firing rate of noise bursts 2–6 in each six-pulse train was compared with the firing rate evoked by the first noise burst. This analysis confirmed that *Cntnap2*^−/−^ multiunit clusters responded significantly poorer than wildtype multiunit clusters to temporally modulated stimuli at the faster repetition rates (7.4 pps: *P_Bonf_* <0.001; 9.2 pps: *P_Bonf_* <0.001; Fig. 6*A*). Furthermore, mean temporal modulation-transfer functions (tMTFs) were created by normalizing the RRTF of each multiunit cluster to the firing rate of the first noise burst in the six-pulse train such that the magnitude of normalized cortical response was defined as a function of the stimulus repetition rate. The tMTF analysis revealed that for both genotypes the cortical responses to modulated stimuli began to degrade when the rate reached 2.8 pps compared with 0.3 pps (wildtype: *P_Dunnet_* <0.001; *Cntnap2*^−/−^: *P_Dunnet_* <0.001; Fig. 6*B*). Finally, to quantify the capacity of cortical neurons to process high-rate stimuli, we calculated the highest temporal rate at which the tMTF of each multiunit cluster was at half its maximum (*f_h1/2_*), and found it to be significantly lower in the *Cntnap2*^−/−^ multiunit clusters compared with wildtypes (*P* < 0.001; Fig. 6*C*). Taken together, these established metrics provide clear evidence that a functional loss of *Cntnap2* results in impairments in auditory temporal processing, characterized by an inability of cortical neurons to sustain their spiking activity to rapidly presented acoustic stimuli.

Finally, the effect of a functional loss of *Cntnap2* on the capacity of cortical neurons to respond in a time-locked fashion to the acoustic stimuli was determined using measures of vector strength and Rayleigh statistics. More specifically, to quantify the temporal fidelity of spikes to the successive noise bursts in the six-pulse train, the vector strength for each multiunit cluster was calculated. Furthermore, Rayleigh statistics were calculated to estimate the significance of vector strength. In general, both the vector strength (Fig. 7*A*) and Rayleigh statistic (Fig. 7*B*) of *Cntnap2*^−/−^ multiunit clusters were greater than that of wildtypes at the repetition rates tested in this study, apart from 9.2 pps (vector strength at 0.3–7.4 pps: *P_Bonf_* <0.001; Rayleigh statistic at 0.3–7.4 pps: *P_Bonf_* <0.01). Thus, the functional loss of *Cntnap2* results in multiunit responses that appear more time-locked to the stimulus presentation. It is worth noting, however, that this outcome may be associated with the lower spontaneous spiking activity of *Cntnap2*^−/−^ multiunit clusters (*P* <0.001, see Fig. 4*B*) as well as the reduced rebound response activity (see Fig. 4*H*) as greater vector strength is associated with lower spontaneous firing rates (Eggermont 1991).

### Postsynaptic Responses Are Typical in the Auditory Cortex of *Cntnap2*  ^−/−^ Rats

In order to further investigate mechanisms underlying the aforementioned changes in cortical neuron activity, *in vitro* whole-cell patch clamp electrophysiology was used. We compared postsynaptic activity of visually identified pyramidal neurons in layers 2/3 of the primary auditory cortex (A1) from adult wildtype and *Cntnap2*^−/−^ rats. Frequency (Fig. 8*B*) and amplitude (Fig. 8*C*) of spontaneous excitatory post-synaptic currents (sEPSCs) were found to be unchanged between the genotypes (wildtype: *n* = 19 neurons from six rats; *Cntnap2*^−/−^: *n* = 13 neurons from four rats). To further investigate synaptic transmission, evoked EPSCs (eEPSCs) were elicited through presynaptic stimulation in layers 5/6 of A1 (Fig. 8*D*) using a paired-pulse stimulation protocol. Assessment of the paired-pulse ratio across the range of ISIs that matched those used to assess multiunit temporal processing (i.e., 108 ms ISI = 9.2 pps, 136 ms ISI = 7.4 pps, 191 ms ISI = 5.2 pps, 358 ms ISI = 2.8 pps; Fig. 8*E*) did not reveal differences between the wildtype and *Cntnap2*^−/−^neurons at any ISI (Fig. 8*F*). Overall, the consistent results in the frequency and amplitude of the sEPSC, as well as in the paired-pulse ratios of eEPSC, suggest that the functional loss of *Cntnap2* did not alter presynaptic input into pyramidal neurons in terms of number of synapses or release probability, nor postsynaptic receptor densities.

#### Auditory Cortex Neurons Lacking *Cntnap2* Require Less Stimulation for Depolarization and Spike Release

To assess the intrinsic cell properties of pyramidal neurons in layers 2/3 of the primary auditory cortex from adult wildtype and *Cntnap2*^−/−^ rats, voltage-clamp configuration was used to measure cell capacitance and membrane resistance, as well as maximum rapid voltage-activated inward current density (presumably sodium currents, normalized to cell capacitance) and current half-width. Furthermore, the resting membrane potential was measured by briefly switching to current-clamp mode. The resting membrane potential was not different between the genotypes (Fig. 9*B*). However, *Cntnap2*^−/−^ neurons had a smaller membrane capacitance (*P* <0.001; Fig. 9*C*), which could be explained by the lower average membrane resistance (wildtype: 44.05 ± 3.75 MΩ vs *Cntnap2*^−/−^: 28.27 ± 13.22 MΩ; *P* = 0.01). The current density of the transient voltage-activated inward current was altered in *Cntnap2*^−/−^ neurons compared with wildtypes (genotype × voltage holding level: *P* = 0.002; Fig. 9*D*). Specifically, *Cntnap2*^−/−^ neurons had larger inward currents across the full range of voltage levels (normalized current at −90—40 mV: *P_Bonf_* <0.001; Fig. 9*D*), which are suggestive of a larger influx of sodium ions during depolarization. The activation threshold of these inward currents was also elicited at a lower voltage in *Cntnap2*^−/−^ neurons (*Cntnap2*^−/−^: −40 mV vs. wildtype: −30 mV; Fig. 9*D*), indicative of a lower threshold for sodium channel activation in cortical pyramidal neurons of *Cntnap2*^−/−^ rats. The half-widths of the inward currents were found to be significantly longer in *Cntnap2*^−/−^ neurons (wildtype: 1.2 ± 0.03 ms vs. *Cntnap2*^−/−^: 1.65 ± 0.07 ms; *P* <0.001). Correspondingly, *Cntnap2*^−/−^ neurons exhibited lower spike firing thresholds (*P* = 0.01; Fig. 9*E*) and lower rheobase currents (*P* = 0.02; Fig. 9*F*) in the current-clamp mode; these findings further suggest an increased intrinsic excitability in pyramidal neurons of the auditory cortex from *Cntnap2*^−/−^ rats.

### Action Potential Features and Spiking Kinetics Are Altered in Cortical Neurons with a Functional Loss of *Cntnap2*

Considering that genotypic differences were observed in the sound-evoked multiunit firing rates *in vivo*, we investigated whether a functional loss of *Cntnap2* altered action potential features and kinetics in the current-clamp mode. Compared with those from wildtype rats, *Cntnap2*^−/−^ neurons had decreased action potential peak amplitudes (*P* = 0.008; Fig. 10*B*) and exhibited longer action potential half-widths (*P* <0.001; Fig. 10*C*). Furthermore, the fast trough of the after-hyperpolarization was not different between genotypes (Fig. 10*D*) whereas the slow trough component was larger in *Cntnap2*^−/−^ neurons (*P* = 0.03; Fig. 10*E*). Despite these differences, the ISIs in response to depolarizing current injections were not significantly different in *Cntnap2*^−/−^ neurons (*P* = 0.07; Fig. 10*F*). Overall, the longer half-width and larger after-hyperpolarization suggest that *Cntnap2*^−/−^ neurons have slower rectifying currents than wildtype neurons. Coupled with the increased excitability (i.e., lower spike threshold), these features of action potentials resulted in an altered firing profile (i.e., significant interaction between genotype × current injection level: *P* = 0.001; Fig. 10*G*). More specifically, at low current injection levels, the *Cntnap2*^−/−^ neurons firing rate (number of spikes in 1 s) was greater than wildtype neurons (e.g., at 0.12 nA: *P*_Bonf_ = 0.05); however, as the current injected was increased, the number of spikes elicited was lower in the *Cntnap2*^−/−^ versus wildtype neurons (e.g., at 0.4 nA: *P*_Bonf_ = 0.03; Fig. 10*G*).

In summary, patch-clamp recordings of pyramidal neurons in the auditory cortex indicate a higher intrinsic excitability of these neurons in *Cntnap2*^−/−^ rats due to lower firing thresholds and changes in current dynamics that impact the neurons’ responses to ongoing depolarizations while there is no evidence for changes in synaptic events.

## Discussion

The present study included a series of experiments to determine whether the association between the *CNTNAP2* gene and language impairment in clinical populations (e.g., ASD and DLD) could potentially arise from its role in cortical auditory processing. Using adult wildtype and *Cntnap2*^−/−^ rats, we investigated the working hypothesis that a constitutive loss of *Cntnap2* function causes altered excitability and poorer temporal processing of sound, similar to what is seen in language-related disorders such as ASD and DLD. As predicted, our findings recapitulated the immature-like CAEP profile in ASD, whereby the sound-evoked N1 potential showed a delayed onset and larger amplitude in knockout compared with wildtype adult rats. Moreover, extracellular electrophysiological recordings of multiunit activity also showed delayed and prolonged responses to a single noise burst, ultimately resulting in a greater response magnitude. Despite this larger response to simple acoustic stimuli, *Cntnap2*^−/−^ rats had a lower capacity to process high-rate temporally modulated stimuli compared with wildtype rats as evidenced by a lower firing rate to rapidly presented stimuli (i.e., RRTF, *f_h1/2_*). Hyperexcitable pyramidal neurons and altered action potential features as shown by our patch-clamp recordings could underlie the changes in multi-unit activity observed in the *Cntnap2*^−/−^ rats. In the following sections, we discuss our collective findings in the context of cortical maturation, excitability, and potential cellular mechanisms.

### Immature-Like Auditory Function

Based on the collective findings from studies that investigated the development of the auditory system in neurotypical and autistic individuals, it has been hypothesized that the maturational stage of the auditory cortex, as measured by CAEP, can be indicative of altered language development and represents a hallmark of language-related disorders (Oram Cardy et al. 2008; Roberts et al. 2010; Edgar et al. 2015; Berman et al. 2016; Kwok, Joanisse, Archibald, Oram Cardy 2018a; Kwok, Joanisse, Archibald, Stothers, et al. 2018b). Similar to the immature-like CAEP profile in the adult *Cntnap2*^−/−^ rats, the multiunit response also had a delayed onset and greater response magnitude. Moreover, the differences in multiunit activity in response to temporally modulated stimuli observed in the adult *Cntnap2*^−/−^rats in the present study are reflective of the profile commonly observed in young wildtype rats. Specifically, the interaction in the RRTF between firing rate and repetition rate, as well as the reduced rate following ability (*f_h1/2_*), and the increased temporal fidelity (VS) observed in *Cntnap2*^−/−^ rats, are shifted in the direction that is observed in younger rats at presentation rates up to 10 pps (Chang et al. 2005). Given that immaturity is reflected in both the N1 potential and the multiunit response profile to temporally modulated stimuli in the auditory cortex of adult *Cntnap2* knockout rats, *CNTNAP2*-mediated differences in language processing could indeed arise from its role in affecting the cortical response to sound.

The immature-like profile of responses in the adult auditory cortex observed in the present study has not been consistently recapitulated in all rodent models for language-related disorders. The prenatally injected valproic acid (VPA) rat (model for ASD) shows the most similar profile to the *Cntnap2*^−/−^ rat. For example, VPA rats exhibited prolonged CAEP latencies (Gandal et al. 2010; Engineer, Centanni, Im, Borland, et al. 2014a), increased N1–P2 amplitudes (Engineer, Centanni, Im, Borland, et al. 2014a; cf. Gandal et al. 2010), and delayed multiunit onset and peak latency in the anterior auditory fields; however, compared with control rats, the VPA rats did not have a greater multiunit firing rate or altered temporal fidelity (Engineer, Centanni, Im, Borland, et al. 2014a). Although the *Kiaa0319* knockdown rats (model for dyslexia) also had a prolonged CAEP latency compared with wildtypes, they had a smaller N1 amplitude and poorer temporal fidelity (Centanni et al. 2014). Likewise, the N1–P2 amplitude is reduced in *Fmr1* knockout rats (model for fragile X syndrome) compared with wildtypes, with no differences in multiunit response characteristics in the primary auditory cortex (Engineer, Centanni, Im, Rahebi, et al. 2014b). That said, similar to the *Cntnap2*^−/−^ rats, the *Fmr1* knockout mouse model does show increased firing rates in response to an acoustic stimulus, and a longer response duration, due to a greater variability in spike latency (Rotschafer and Razak 2013).

Rat models based on early-life perturbations that are relevant for human development also show immature-like response profiles to temporally modulated stimuli. For example, in humans, lead exposure is a risk factor for learning disability, and prenatal lead exposure in rats leads to a decreased cortical capacity for processing high-rate acoustic stimuli (Zhu et al. 2016). Pulsed noise exposure during a critical period for auditory development similarly leads to poorer processing of temporally modulated stimuli at rates greater than approximately 9 pps, a slower maximum rate following ability (i.e., *f_h1/2_*), as well as increased temporal fidelity at rates up to 9 pps (Zhou and Merzenich 2008); these findings are similar to those observed in the *Cntnap2*^−/−^ rats.

### Excitability

To determine whether the altered temporal processing observed in the *Cntnap2* knockout rats resulted from differences in cortical excitability, we assessed the postsynaptic activity and intrinsic membrane properties of pyramidal neurons in the auditory cortex using *in vitro* whole-cell patch clamp electrophysiology. We found no evidence for changes in presynaptic input to *Cntnap2*^−/−^ cells. The amplitude and frequency of spontaneous synaptic events were the same, and paired pulse ratios, a common measure of presynaptic transmitter release probability, were not different between genotypes. That said, because no pharmacological manipulations were used to separate excitatory from inhibitory input, it cannot be ruled out that the proportion of inhibitory versus excitatory input is changed. Although the lack of genotypic differences in the immunohistochemical staining for cellular markers associated with glutamatergic and GABAergic neurotransmission supports the suggestion that presynaptic input to *Cntnap2*^−/−^ cells is not altered (see Supplementary Figure 1).

In contrast, intrinsic membrane properties of pyramidal neurons differed between *Cntnap2*^−/−^ and wildtype cells. Fast transient inward currents (presumably sodium currents) were activated at lower voltages and lasted for longer periods of time (i.e., larger half-width) in *Cntnap2*^−/−^ cells. This was also reflected by lower spike thresholds in the current-clamp modus. Lower activation thresholds were observed despite lower membrane resistance in knockout cells, which would expect to increase, not decrease, the rheobase due to a lower input resistance of the cell. Our observations therefore indicate a higher excitability of *Cntnap2*^−/−^ cells, presumably due to changes in expression and/or function of voltage-gated ion channels. Furthermore, the spike activity profile of pyramidal neurons was also different in *Cntnap2*^−/−^ neurons dependent on the extent of depolarization: Knockout cells produced more spikes than wildtype cells at a low current stimulation level but fewer spikes at stronger depolarization levels. Together with the observed changes in the after-hyperpolarization, this again indicates differences in voltage-gated ion channel expression or function, most likely in voltage- and calcium-activated potassium channels that are responsible for repolarization of the membrane.

Although the present study is the first to examine the role of *Cntnap2* in the excitability of neurons in the auditory pathway, action potential features have been studied previously in other sensory areas in mouse models of *Cntnap2* dysfunction. For example, greater spike rates were found in layer 4 of the somatosensory cortex of *Cntnap2*^−/−^ mice regardless of current injected (Antoine et al. 2019) whereas another study showed no differences in firing threshold, spike amplitude, spike width, or firing rate in layers 2/3 of the visual cortex of *Cntnap2*^−/−^ mice (Bridi et al. 2017). Given that our data suggests specific changes in voltage-gated ion channels that are responsible for altered excitability in the auditory cortex of *Cntnap2*^−/−^ rats, it might be worthwhile for future studies to further investigate these changes in the neurons of the auditory, visual, and somatosensory cortices in a model of *Cntnap2* dysfunction.

### Potential Cellular Mechanisms

Taken together, the increased multiunit firing rate observed in the *Cntnap2*^−/−^ rats *in vivo* could directly arise from the altered excitability and spiking profile of pyramidal neurons observed *in vitro*, leading to the acoustic stimulus exciting a greater number of neurons and each neuron firing more action potentials if a single noise burst is equivalent to a low level of stimulation. Once the capacity of the auditory cortex to respond to temporally modulated stimuli was tested by presenting pulse trains consisting of six noise bursts presented at rapid repetition rates (e.g., 9.2 pps), the *Cntnap2*^−/−^ multiunit firing rate decreased, akin to the reduced ability of knockout pyramidal neurons to spike at high stimulation currents. Therefore, the hyperexcitability of pyramidal neurons and spiking profile could explain the differences in multiunit activity to both temporally simple and complex acoustic stimuli, which could likely be due to changes in the expression and/or function of ion channels. However, as mentioned above, changes in glutamatergic versus GABAergic neurotransmission as observed in ASD and DLD cannot be completely ruled out. It is worth noting that rodent studies that used pharmacological manipulations in the auditory cortex to either increase local glutamatergic activity via glutamate application, or reduce GABAergic activity via GABA antagonists (Chang et al. 2005; Kurt et al. 2008; Moeller et al. 2010; Beh 2017), have found a pattern of electrophysiological responses akin to what we have observed in the *Cntnap2* knockout rats (i.e., increased N1 CAEP amplitude, increased multiunit firing rates). Perhaps increased excitability, regardless of the mechanism, can give rise to the observed CAEP and multiunit profile and could provide a common method through which several underlying mechanisms lead to altered cortical responses to temporally modulated stimuli.

### *Cntnap2* and Altered Development

Here, we have shown that a constitutive loss of *Cntnap2* gene function leads to altered pyramidal neuron excitability, poorer temporal control over neural activity, and a profile of immature-like cortical auditory processing in adulthood. These findings are largely consistent with past studies of altered development of the auditory system. For example, depriving the developing auditory system of normal acoustic experience (by rearing animals in a noisy environment, Zhou and Merzenich 2008) caused the same pattern of auditory processing differences in adulthood as observed in the present study. Moreover, gerbils with transient hearing loss during the critical period for auditory development showed an *in vitro* action potential profile similar to what is found in *Cntnap2*^−/−^ rats, including smaller action potential amplitudes, increased half-widths, and reduced firing rate (Mowery et al. 2015). In considering the developmental trajectory of auditory processing associated with *Cntnap2*, we have previously shown that there is a delayed maturation of the brainstem auditory-evoked potential in *Cntnap2*^−/−^  *rats* early in life that normalizes by adulthood (Scott et al. 2018). Given that the brain undergoes extensive remodeling and plasticity during this time of early development, and the interruption of typical maturation during this critical period can lead to long-term auditory processing deficits that persist into adulthood (Sinclair et al. 2016), it is reasonable to propose that the *Cntnap2*-related alteration of sensory input to the auditory cortex during development could cause the persistence of cortical hyperexcitability and profile of immaturity in adulthood. With respect to the possible cellular mechanisms underlying these persistent cortical alterations, it is worth noting that a loss of function of the *Cntnap2* protein, CASPR2, has been shown to block experience-dependent homeostatic synaptic plasticity in the visual cortex in response to dark rearing (Fernandes et al. 2019). Ultimately, the loss of *Cntnap2* seems to lead to long-term cortical auditory dysfunction due to changes in differential neuronal homeostatic plasticity during development, brought on by delayed maturation of sound processing in the brainstem early in life and the prevailing loss of *Cntnap2* function.

### Experimental Limitations and Future Considerations

Overall, our collective results demonstrate that a constitutive loss of the *Cntnap2* gene may contribute to the maintenance of neuronal excitability within the cortex and underlie some of the cortical alterations observed in *Cntnap2*^−/−^ rats. However, there are a few experimental limitations in the present study that should be addressed and considered in future research to provide a comprehensive understanding of the contributions of the *Cntnap2* gene on auditory processing. For example, although the present study utilized a dorsomedial-to-ventrolateral recording approach in order to preserve auditory cortex function during the recording session, this limited our ability to precisely record across the cortical layers. As previous studies in mice lacking the *Cntnap2* gene have shown deficits in the migration of cortical projection neurons resulting in an abnormal clustering of neurons in the deep layers of the cortex (Peñagarikano et al. 2011), future studies should consider examining auditory responsiveness across the cortical layers to provide insights into alterations in the cortical microcircuit within the auditory cortex in rodent models of ASD and DLD.

Given that a knockout of *Cntnap2* in mice resulted in altered auditory-related behaviors, such as enhanced pitch discrimination and deficits in temporal processing (Truong et al. 2015), it would be worthwhile for future studies to conduct a thorough examination of the morphological and physiological properties of the auditory cortex in rodent models of ASD. For example, a multishank electrode could be used to map the tonotopy of the auditory cortex in *Cntnap2*^−/−^ rodents. This would potentially provide a neural correlate for the enhanced tone discrimination and pitch perception reported in *Cntnap2*^−/−^ mice. Moreover, the altered neuronal response properties observed in the present study could be correlated with the neuron’s characteristic frequency so as to determine whether the observed alterations occur across the entire auditory cortex or are restricted to a specific frequency range of the tonotopic map.

Finally, as the present study focused on examining alterations in auditory temporal processing at rates that show consistent evoked activity across most of the pulses in the stimulus train (i.e., rates below 9 pps), it is important that future studies examine the ability of the cortex in *Cntnap2*^−/−^ rats at higher repetition rates as this would provide insights into the severity of auditory temporal processing in this rodent model of ASD and DLD. Additional temporal processing metrics, such as speech sounds and gap detection in quiet and noise, should also be examined as rodent models of ASD have shown behavioral alterations in these auditory processing tasks (Truong et al. 2015). Overall, the results of the present study provide evidence of auditory processing impairments in response to simple and complex acoustic information, with the hope of future studies further examining the mechanisms underlying these alterations.

## Conclusion

Overall, our findings revealed immaturity (i.e., poor temporal resolution) and hyperexcitability in the auditory cortex of adult *Cntnap2* knockout rats. The immature-like CAEP and multiunit temporal processing profiles observed in adulthood further validate the *Cntnap2* knockout rat model for studying auditory system dysfunction with high relevance to language-related disorders, including ASD and DLD. The pattern of hyperexcitability and action potential kinetics in pyramidal cell recordings suggest that the *Cntnap2* gene is involved in maintaining cortical excitability. Future studies using rodent models with a functional loss of the *Cntnap2* gene could examine the developmental changes in homeostatic plasticity and circuit function in the auditory cortex to ultimately determine the role of *Cntnap2* in central auditory processing across age.

## Supplementary Material

Supplementary_file_bhab517

## Notes

We would like to acknowledge Alisha Moosajee for assistance with immunohistochemistry. *Conflict of Interest*: None declared.
