# Supplementary material for: Hyperexcitable and immature-like neuronal activity in the auditory cortex of adult rats lacking the language-linked CNTNAP2 gene
Source: Cereb Cortex. 2022 Feb 1;32(21):4797–817. doi: 10.1093/cercor/bhab517 (PMC9626820; doi:10.1093/cercor/bhab517)
Supplement: Supplementary_file_bhab517 [file supplementary_file_bhab517.docx]

**Suppl. Fig. 1:**


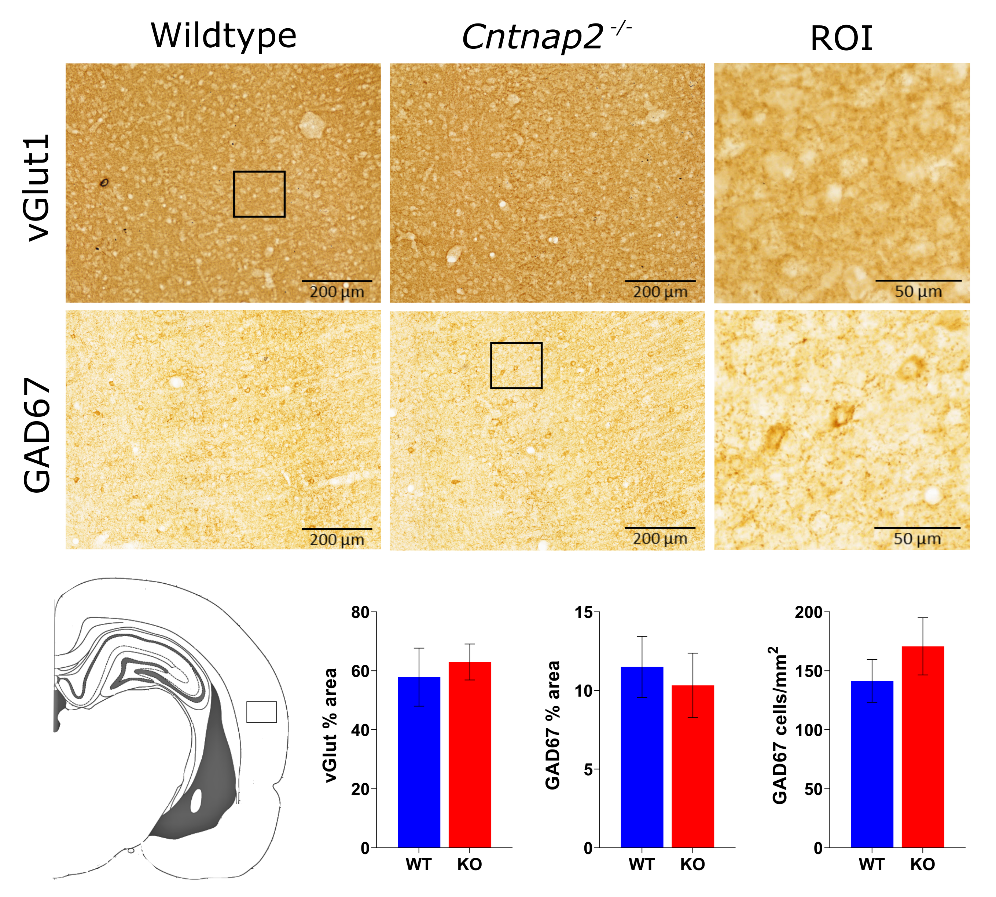
**VGlut1 and GAD67 immuno-reactivity does not differ from wildtypes in *Cntnap2-/-* rats**

***Top*:** Representative images of the immunoreactivity to the glutamatergic marker vesicular glutamate transporter 1 (VGlut1) and the GABAergic marker glutamic acid decarboxylase 67 (GAD67) in the auditory cortex (see rectangle on coronal section schematic) of a wildtype and

*Cntnap2-/-* rat. Representative ROI images, taken at 20X magnification, are outlined by small black squares. ***Bottom:*** Quantitative analysis of VGlut1 and GAD67 showing the total percentage of area stained, and the number of GAD67 stained cell bodies (wildtype n = 6,

*Cntnap2-/-* n = 7). Data expressed as mean ± SEM.

**Appendix A: Immunohistochemistry**

**Materials and Methods**

*Immunohistochemistry*

The opposing auditory cortex of select animals in which *in vivo* electrophysiology was completed were used to stain for GAD67 (wildtype, n = 7; *Cntnap2-/-*, n = 6) and VGluT1 (wildtype, n = 7; *Cntnap2-/-*, n = 6). Before free-floating immunohistochemistry, as well as in between all incubations with antibodies, all slices were thoroughly rinsed in 0.1 M phosphate buffered saline (PBS). Slices were pretreated with a 1% H2O2 in 0.1 M PBS for 10 min. Sections were then blocked in a PBS solution containing 0.2% Triton-X (Thermo Fisher Scientific, Waltham, MA) and 0.1% bovine serum albumin (Sigma-Aldrich, St. Louis, MO) for 1 hour, before incubation in the blocking solution with the primary antibody, either mouse anti-GAD67 primary antibody (1:5000; MAB5406, Millipore, USA) or guinea pig anti-VGluT1 primary antibody (1:5000; AB5905, Sigma-Aldrich, St. Louis, MO) overnight at room temperature. Next, sections were incubated in the blocking solution with biotinylated secondary antibody (1:500; anti-mouse, BA-9200, Vector Labs, Burlingame, CA or 1:500; anti-guinea pig, 106-065-003, Jackson ImmunoResearch Lab; West Grove, PA) for 1 hour at room temperature, then processed using avidin-biotin complex solution (1:1000; Vectastain Elite ABC Kit; pk 6100; Vector Labs, Burlingame, CA) in PBS for 1 hour at room temperature. Labelling was visualized using 3,3’-diaminobenzidine (DAB) solution (0.04% H2O2, 0.2 mg/mL DAB; D4293, Sigma-Aldrich, St. Louis, MO) at room temperature. The slices being stained for GAD67 were incubated for 7 min, the slices being stained for VGluT1 were incubated for 3 min. Tissue was then mounted onto positively charged glass slides, air dried overnight, dehydrated in increasing concentrations of alcohol, cleared in xylene, and coverslipped with DPX mounting media (HX55746679, Millipore, USA).

*Imaging and processing*

Imaging was performed using a Nikon Eclipse Ni-U upright microscope with a DS-Qi2 high definition color camera and imaging software NIS Elements Color Camera (Nikon Instuments Inc., Melville, NY). The complete auditory cortex in a given slice was imaged by capturing a stitched image at 10x (VGluT1) or 20x (GAD67) magnification. Images were then processed using Fiji (Schindelin et al., 2012). Using rectangular rulers scaled via the specify tool, the auditory cortex (region of interest, ROI) was defined as the region spanning 1500 – 2500 μM dorsal to the rhinal fissure, from the inner edge of the cortex to the outer edge and outlined using the polygon tool. Images were converted to 8-bit, processed using the despeckle command, and then thresholded with a fixed grayscale cut-off value. Protein expression was determined by calculating the staining intensity (% area coverage), and density (cells/mm2) within the auditory cortex (Suppl. Fig. 1). Using the ROI manager, area coverage (%) and total area (mm2) were measured and recorded for each ROI. Cell count was determined using the analyze particles tool, and was divided by the total ROI area. Data analyses were performed with Fiji (Schindelin et al., 2012) and Microsoft Excel 2010 (Microsoft Corp.).

*Statistics*

To compare genotypic differences in the expression of excitatory and inhibitory markers, independent samples t-tests were performed for VGluT1 percent area coverage, GAD67 percent area coverage, and the number of GAD67 reactive cell bodies.

**Results**

*A functional loss of Cntnap2 did not affect the expression of VGluT1 or GAD67 in the auditory cortex*

To investigate whether the changes in pyramidal neuron excitability is due to intrinsic membrane properties or might be due to changes in tonic inhibition by GABA, we assessed the effect of a functional loss of *Cntnap2* on cellular markers associated with excitatory and inhibitory neurotransmission by comparing the expression of VGluT1, a glutamate transporter associated with the synaptic vesical membrane, as well as GAD67, a key synthesizing enzyme for GABA found throughout the cell, in the auditory cortex of *Cntnap2-/-* (n = 7) and wildtype (n = 6) rats. As shown in Supplemental Figure 1, there was no genotypic difference in the percent area coverage of VGluT1 (*p* = 0.65; top panels) or GAD67 staining (*p* = 0.69; bottom panels). Moreover, as GAD67 also stains the cell body, we further compared the number of immune-reactive cell bodies in the auditory cortex of the *Cntnap2-/-* versus wildtype rats, ultimately finding no differences between genotypes (*p* = 0.34).

**Appendix B: Statistics Table 1**
